# Supplementary material for: Tailocin tail fiber diversity correlates with rfbD variation in the Pseudomonas syringae species complex
Source: ISME Commun. 2025 Jun 12;5(1):ycaf099. doi: 10.1093/ismeco/ycaf099 (PMC12208367; doi:10.1093/ismeco/ycaf099)
Supplement: Supplementary_Materials_ycaf099 [file supplementary_materials_ycaf099.pdf]

Supplementary Material for

Tailocin Tail Fiber Diversity Correlates with rfbD Variation in The *Pseudomonas syringae* Species Complex

Chad Fautt[1,3]; Kevin Hockett[2,3] Simon Delattre[4]; David Baltrus[5,6;] Estelle Couradeau[1,3]

1. Department of Ecosystem Science and Management, Pennsylvania State University, University Park, Pennsylvania, United States

2. Department of Plant Pathology and Environmental Microbiology, Pennsylvania State University, University Park, Pennsylvania, United States

3. Intercollege Graduate Degree Program in Ecology, Pennsylvania State University, University Park, Pennsylvania, United States

4. Institute for Computational and Data Sciences, Pennsylvania State University, University Park, Pennsylvania, United States

5. School of Plant Sciences, University of Arizona, Tucson AZ , USA

6. School of Animal and Comparative Biomedical Sciences, University of Arizona, Tucson AZ ,

USA

*Corresponding authors:

Estelle Couradeau

450 ASI

University Park, PA 16802

United States

efc5279@psu.edu

Kevin Hockett

316 Buckhout Lab

University Park, PA 16802

United States

klh450@psu.edu

Description of generated data

(deposited at https://doi.org/10.5281/zenodo.10035485)

Supplementary data 1

HMM_1, representing tailocin tail fibers associated with killing class 1

Supplementary data 2

HMM_2, representing tailocin tail fibers associated with killing class 2

Supplementary data 3

HMM_3, representing tailocin tail fibers from PSSC strain UB246

Supplementary data 4

Amino acid sequence for WP_044313553.1, representative of type 1a tailocin-associated tail fiber used for protein structure prediction in Supplementary data 5

Supplementary data 5

PDB file containing predicted structure of WP_044313553.1, representative of type 1a tailocin-associated tail fiber

Supplementary data 6

Amino acid sequence for WP_122688044.1, representative of type 1b tailocin-associated tail fiber used for protein structure prediction in Supplementary data 7

Supplementary data 7

PDB file containing predicted structure of WP_122688044.1, representative of type 1b tailocin-associated tail fiber

Supplementary data 8

Amino acid sequence for WP_005768002.1, representative of type 2 tailocin-associated tail fiber used for protein structure prediction in Supplementary data 9

Supplementary data 9

PDB file containing predicted structure of WP_005768002.1, representative of type 2 tailocin-associated tail fiber

Supplementary data 10

Amino acid sequence for WP_024674765.1, representative of type 3 tailocin-associated tail fiber used for protein structure prediction in Supplementary data 11

Supplementary data 11

PDB file containing predicted structure of WP_024674765.1, representative of type 3 tailocin-associated tail fiber

Supplementary data 12

Amino acid sequence for WP_198721597.1, representative of RSA1-like prophage-associated tail fiber used for protein structure prediction in Supplementary data 13

Supplementary data 13

PDB file containing predicted structure of WP_198721597.1, representative of RSA1-like prophage-associated tail fiber

Supplementary data 14

CSV file containing HMM_1 and HMM_2 genomic screen results, with accession numbers and identities of tail fibers detected in each genome.

Supplementary data 15

CSV file containing HMM_3 genomic screen results, with copy number of tail fibers detected in each genome and the phylogroup the genome belongs to

Supplementary Figures

Fig. S1

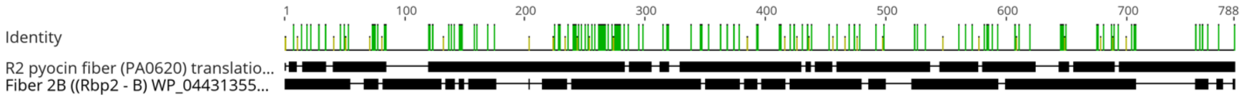

Pairwise alignment of type 1a tailocin-associated tail fiber found in PSSC and an R2 pyocin fiber from *P. aeruginosa*

Fig. S2

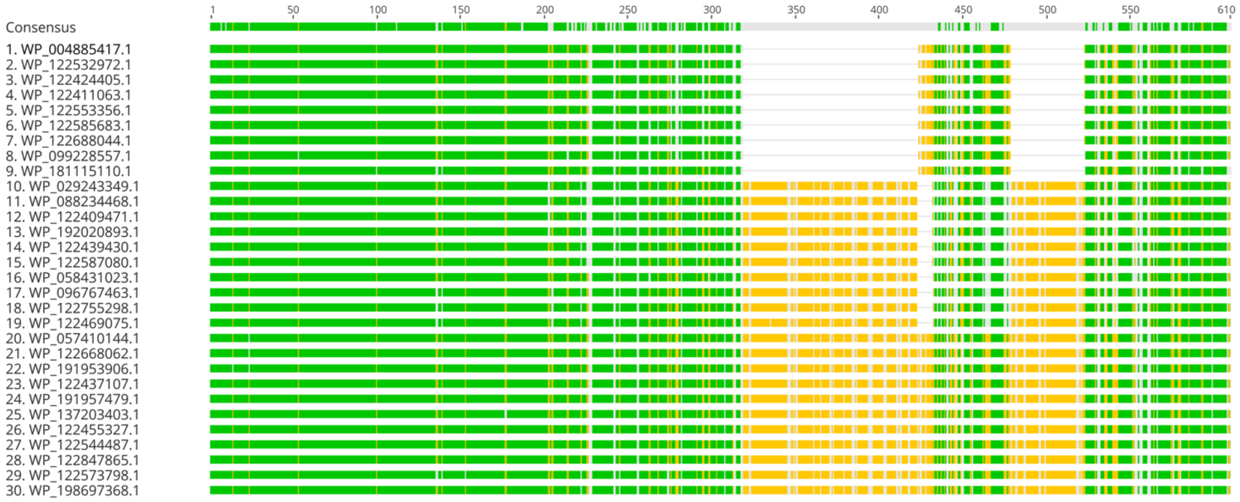

Multiple amino acid sequence alignment for all unique type 1b fibers found in this study

Fig. S3

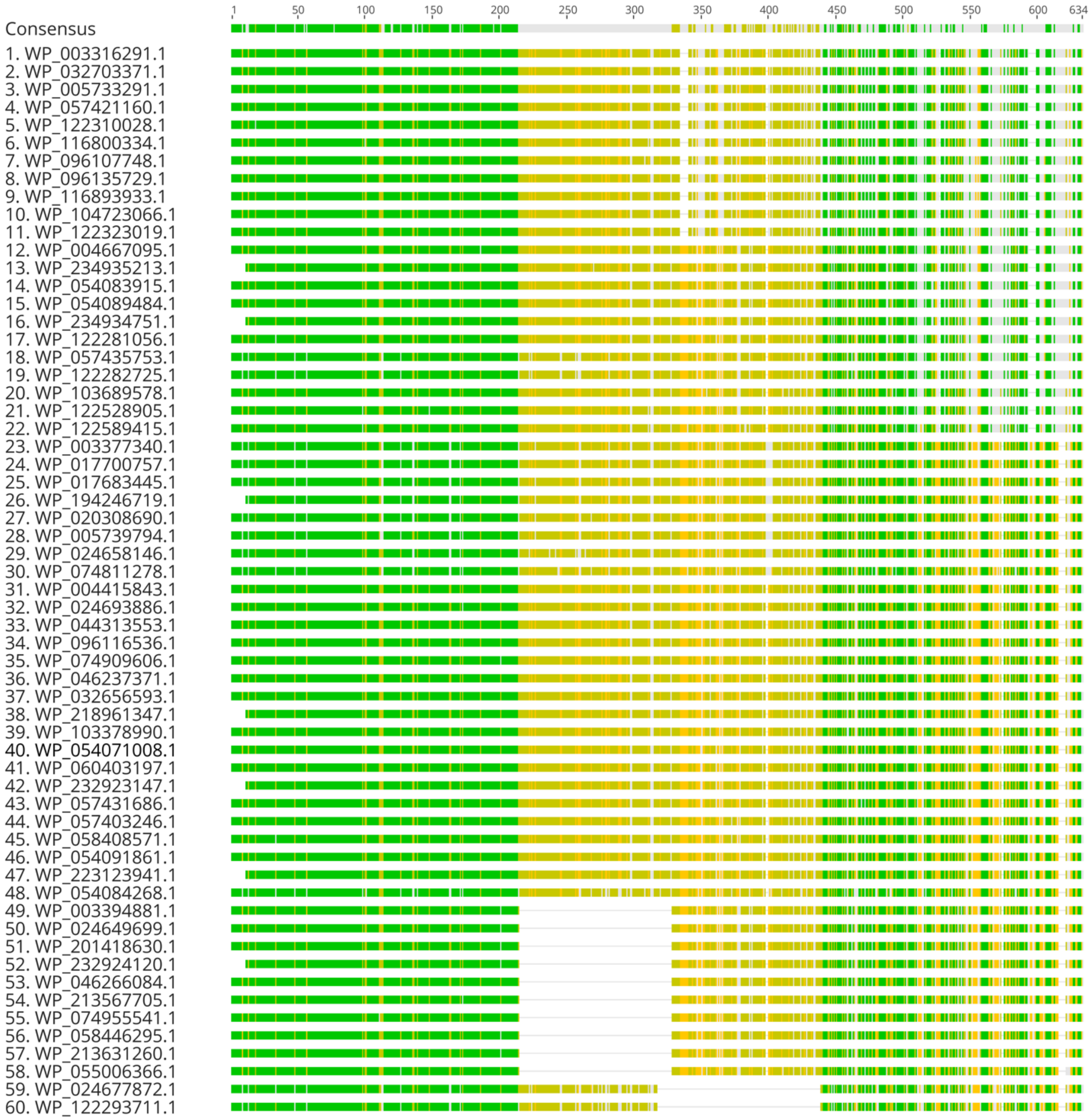

Multiple amino acid sequence alignment for all unique type 1a fibers found in this study
